# SORDOR pulses: expansion of the Böhlen–Bodenhausen scheme for low-power broadband magnetic resonance

**DOI:** 10.5194/mr-3-53-2022

**Published:** 2022-04-27

**Authors:** Jens D. Haller, David L. Goodwin, Burkhard Luy

**Affiliations:** 1 Institute for Biological Interfaces 4 – Magnetic Resonance, Karlsruhe Institute of Technology (KIT), Karlsruhe, Germany; 2 Institute of Organic Chemistry, Karlsruhe Institute of Technology (KIT), Karlsruhe, Germany

## Abstract

A novel type of efficient broadband pulse, called second-order phase dispersion by optimised rotation (SORDOR), has recently been introduced. In contrast to adiabatic excitation, SORDOR-90 pulses provide effective transverse 90 ${}^{\circ }$ rotations throughout their bandwidth, with a quadratic offset dependence of the phase in the x,y plane. Together with phase-matched SORDOR-180 pulses, this enables the Böhlen–Bodenhausen broadband refocusing approach for linearly frequency-swept pulses to be extended to any type of 90 ${}^{\circ }$ /180 ${}^{\circ }$ pulse–delay sequence. Example pulse shapes are characterised in theory and experiment, and an example application is given with a ${}^{19}F$ -PROJECT experiment for measuring relaxation times with reduced distortions due to J -coupling evolution.

## 1 Introduction

Many magnetic resonance applications require the manipulation of spins over a large bandwidth under severe restrictions concerning available radiofrequency (rf) amplitude and/or tolerable rf energy. With the advent of 1.2 GHz NMR spectrometers, a large number of experiments require the use of amplitude- and phase-modulated pulses due to the enlarged bandwidths that need to be covered, and equally modern pulsed EPR spectroscopy at any field strength benefits substantially from the use of shaped pulses [Bibr bib1.bibx22]. However, even conventional NMR spectroscopy at intermediate field strengths at e.g. 600 MHz gain enormously from shaped pulses in conventional carbon-correlated experiments [Bibr bib1.bibx75] and even more so with particular nuclei like ${}^{19}F$ , ${}^{31}P$ , ${}^{15}N$ , ${}^{119}\mathrm{Sn}$ , and ${}^{195}\mathrm{Pt}$ [Bibr bib1.bibx32].

A multitude of composite and shaped pulses has been designed to cope with the bandwidth problem [Bibr bib1.bibx66], with recent pulse shapes optimised using algorithms derived from optimal control theory [Bibr bib1.bibx18] being close to physical limits [Bibr bib1.bibx56]. Whenever only a single component of magnetisation vectors needs to be transferred, excitation and inversion pulses, as members of *point-to-point* (PP) pulses, provide very efficient solutions [Bibr bib1.bibx82]; regardless, only *universal rotation* (UR) pulses can be used as full replacements of conventional, bandwidth-limited, hard pulses. UR pulses, however, are especially demanding regarding both rf amplitude and rf energy [Bibr bib1.bibx96].

To reduce such demands, several concepts involving matching pulse shapes have been developed. Possibly the first such concept based on adiabatic pulses has been reported by Böhlen and Bodenhausen, requiring matched, linear-frequency-swept excitation and inversion pulses [Bibr bib1.bibx9]. Depending on the offset $\omega_{z}$ , adiabatic excitation transforms z magnetisation into transverse magnetisation with pulse-dependent phase angles $\alpha (\omega_{z})$ with respect to the x axis. A following matched adiabatic inversion pulse provides an effective rotation around the phase $\alpha (\omega_{z})+\varphi$ with either constant or linearly offset-dependent phase φ . As the effective pulse phases are matched in this concept, the inversion pulses act as refocusing (UR-180) pulses whose effective rotation axes exhibit a quadratic offset dependence due to the adiabatic linear frequency sweep. A more general concept without the restriction on linear frequency sweeps is the COOP concept introduced by Braun and Glaser [Bibr bib1.bibx11]. However, here too excitation pulse shapes introduced so far are PP pulses, resulting in limited applicability.

Here we propose the recently introduced *second-order phase dispersion by optimised rotation* (SORDOR) pulses of [Bibr bib1.bibx45] for an extension of the Böhlen–Bodenhausen concept for a widely applicable cooperative broadband-shaped pulse scenario, in which 90 ${}^{\circ }$ universal rotations are directly realised. SORDOR pulses are not adiabatic and define a new class of low-energy pulse shapes that cause a defined rotation with a constant rotation angle over a specified bandwidth like common UR pulses. Instead of the constant rotation axes of conventional UR pulses, SORDOR pulses show a quadratic offset-dependent phase change of the effective rotation axes in the x,y plane, similar to the linear adiabatic sweeps in the Böhlen–Bodenhausen concept. If a simple quadratic-phase correction of spectra can be applied, SORDOR pulses can be used as a direct replacement of 90 and 180 ${}^{\circ }$ hard pulses. In addition, scaled SORDOR-180 pulses allow full refocusing of magnetisation. The SORDOR pulses therefore represent a direct implementation of the Böhlen–Bodenhausen concept of matched quadratic-phase pulses to the requirements of 90 ${}^{\circ }$ -based mixing. After an introduction of pulse properties, their application in NMR spectroscopy is demonstrated experimentally.

## 2 Theory

SORDOR pulses used here have been optimised as described in the reference [Bibr bib1.bibx45]: effective propagation for a single spin 1/2 is given by the n piecewise constant elements of the pulse shape as $U_{\mathrm{eff}}=U_{n}\ldots U_{1}$ , which is subsequently used to calculate a quality factor

1 Φ=Re〈Ueff|UT〉,

where the target propagator is defined by

2 $$U_{T}=\mathrm{exp}\left(-i\beta \left[\mathrm{cos}\left(\alpha (\omega_{z})\right)I_{x}+\mathrm{sin}\left(\alpha (\omega_{z})\right)I_{y}\right]\right),$$

with

3 $$\alpha (\omega_{z})=Q\frac{t_{p}}{2\Omega }{\omega_{z}}^{2}+\alpha_{0},$$

where β defines the desired effective flip angle, α a phase in the x,y plane, $\omega_{z}$ the offset, $I_{x}$ and $I_{y}$ the usual spin operators represented by the corresponding Pauli matrices, the desired offset range Ω , the pulse length $t_{p}$ , an arbitrary scaling factor |Q|<1 , and an arbitrary constant phase $\alpha_{0}$ . Note that the entire shaped pulse is condensed to a single term within the effective propagator and, hence, it directly describes the effective rotation induced by the shaped pulse.

It is evident from the target propagator that a uniform rotation angle β is targeted, resulting in a pulse class termed *B1 pulses* (not to be mistaken with B ${}_{1}$ fields), as defined originally by [Bibr bib1.bibx67] (an extensive description of different pulse classes is given in the Supplement). However, in addition to the minimum requirement of class B1 pulses, an offset-dependent target phase for the effective rotation axes in the x,y plane is applied. Such a phase was originally introduced with *ICEBERG pulses* of [Bibr bib1.bibx41], but, in contrast to the linear ICEBERG phase, the SORDOR target phase varies with a quadratic function of the offset $\omega_{z}$ . Other phase-dependent control targets were investigated more recently by [Bibr bib1.bibx3] and [Bibr bib1.bibx19]. The quadratic SORDOR phase resembles the quadratic phase being acquired in linear-frequency-swept adiabatic inversion pulses [Bibr bib1.bibx8], which can be used advantageously, as will be shown below.

Using the GRAPE algorithm [Bibr bib1.bibx55] with exact gradients [Bibr bib1.bibx99] as discussed in detail in [Bibr bib1.bibx45], pulses for β=90 ${}^{\circ }$ and β=180 ${}^{\circ }$ were obtained, named SORDOR-90 and SORDOR-180, respectively. While the optimisation of SORDOR-180 pulses turned out to be straightforward, good SORDOR-90 pulses were only obtained after introducing a morphing procedure in the optimisation algorithm (morphic-GRAPE), in which the scaling factor Q and the pulse length $t_{p}$ are slowly adjusted from extrema.

The morphic-GRAPE procedure to produce the SORDOR pulses of [Bibr bib1.bibx45] used four *directional morphs* to produce high-fidelity 90 and 180 ${}^{\circ }$ pulses. The highest-fidelity pulses for a bandwidth of 40 kHz, unsurprisingly, were at the longest durations, $t_{p}=450$ µ s, with rf amplitudes of 10 kHz. Following on from those optimisations, the SORDOR pulses used in this work introduce an additional directional morph stage: a *ramping* stage to increase the desired bandwidth to Ω=50 kHz. To this end, the SORDOR pulses ramp 450 µ s pulses from 40 to 50 kHz, over Q=[0.70,0.71,…,0.85] (these Q values are used as the high-performance SORDOR pulses lay in this range), in increments of 1 kHz.

Increasing the desired bandwidth lowers the pulse performance Φ . To gain higher performance, the SORDOR pulses ramped to Ω=50 kHz are further expanded to longer pulse durations from 450 to 750 µ s, over the best-performing Q from the ramped stage: Q=[0.78,0.79,0.80,0.81,0.82] . The best-performing SORDOR pair, at a nominal pulse duration $t_{p}=720$ µ s, occurred at Q=0.80 , and this pulse pair was further optimised for ±5 % B ${}_{1}$ inhomogeneity over a Gaussian distribution of rf-amplitude multipliers.

**Figure 1 Ch1.F1:**
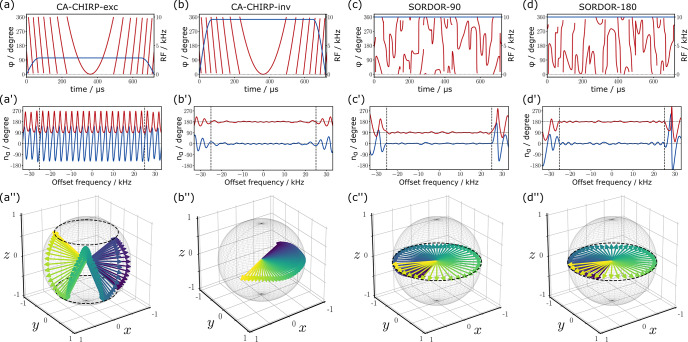
Comparison of a constant adiabaticity chirped excitation pulse (CA-Chirp-exc), a chirped inversion pulse (CA-Chirp-inv), a SORDOR-90, and the matched SORDOR-180 pulse. **(a–d)** Pulse rf amplitudes and phases. **(a'–d')** Offset dependencies of the $\sqrt{x^{2}+y^{2}}$ (red) and z components (blue) of the effective rotations for the specific pulses. **(a”–d”)** Visualisations of the normalised rotational axes of the different pulses for a reduced offset region. Colour coding is used to indicate equal offset frequencies for respective pairs of CA-Chirp and SORDOR pulses. Note that the Chirp excitation pulse does not perform a direct universal 90 ${}^{\circ }$ rotation, while SORDOR-90 does.

The resulting SORDOR-90 and SORDOR-180 pulse pair with identical phase dependence $\alpha (\omega_{z})$ is shown in Fig. [Fig Ch1.F1] together with constant-adiabaticity chirped excitation and inversion [Bibr bib1.bibx53] pulses (CA-Chirp-exc and CA-Chirp-inv) for comparison. The SORDOR pulse shapes have a constant rf amplitude of 10 kHz and in both cases a smooth rf phase, even if the course of the rf phase looks somewhat erratic. The CA-Chirp-exc pulse, instead, has a lower rf amplitude of only 2850 Hz and the well-known smooth rf-phase behaviour. The reason for the difference in rf-energy consumption of the SORDOR-90 and CA-Chirp-exc pulse can be seen in the analysis of resulting effective rotations (Fig. [Fig Ch1.F1]): while the SORDOR-90 at all offsets essentially resembles pure 90 ${}^{\circ }$ rotations with rotational axes in the x,y plane, the CA-Chirp-exc pulse resembles a classical PP excitation pulse with effective rotations in the range of 90–270 ${}^{\circ }$ and a periodic elevation of the rotational axes along z with the offset. This is the typical behaviour of a class B2 pulse, which is explained in more detail in the Supplement. The SORDOR-180 pulse shows the very same rotation behaviour as the SORDOR-90 pulse, just with an effective 180 ${}^{\circ }$ rotation angle in the x,y plane throughout the optimised offset range. As such it resembles very much the behaviour of the CA-Chirp-inv pulse of the same duration and rf amplitude. However, the SORDOR-180 pulse is directly matched in its phase behaviour to the SORDOR-90 pulse, which for an adiabatic inversion pulse would only accidentally be the case.

A systematic study of SORDOR-90 and SORDOR-180 pulse performances Φ for optimal Q values around 0.75 and a bandwidth of 40 kHz as reported in the reference [Bibr bib1.bibx45] is shown in Fig. [Fig Ch1.F2]: with increasing pulse lengths the logarithmic quality factor for pulse performance steadily increases in both cases. As in previous studies exploring the physical limits [Bibr bib1.bibx56], the increase does not describe a smooth function, but as a guide to the eye the pulse performance can be roughly described by a linear function (see Fig. [Fig Ch1.F2]). Also, BURBOP-90 and BURBOP-180 pulses, which can be considered special SORDOR pulses with Q=0 , follow a similar curve [Bibr bib1.bibx59], but for the same performance approximately twice the amount of rf energy is needed for the 180 ${}^{\circ }$ case and about a factor of 1.8 for the 90 ${}^{\circ }$ case. The energy consumption of SORDOR-180 pulses therefore resembles that of time-optimal broadband inversion pulse (BIP) and broadband inversion by optimised pulses (BIBOP) inversion pulses [Bibr bib1.bibx90], while the energy consumption of SORDOR-90 pulses lies in between time-optimal broadband excitation by optimised pulses (BEBOP) excitation and broadband universal rotation by optimised pulses (BURBOP) universal rotation 90 ${}^{\circ }$ pulses.

**Figure 2 Ch1.F2:**
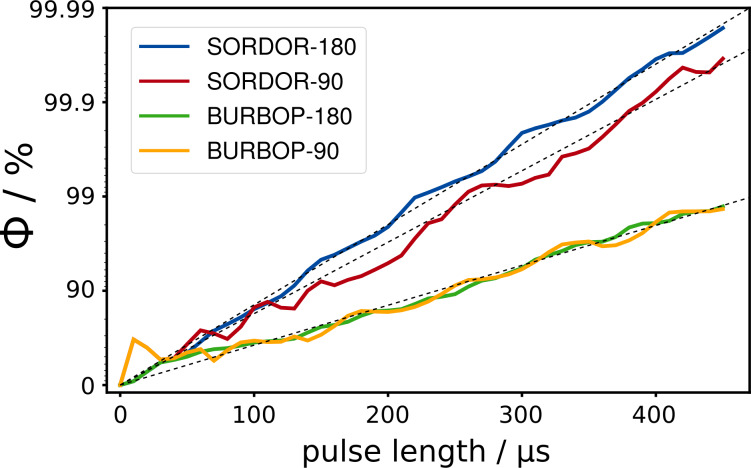
Offset-averaged quality factors Φ for different optimised SORDOR and BURBOP pulse shapes with rf amplitudes of 10 kHz and optimised bandwidths of 40 kHz. SORDOR-180 pulses of the same quality consume approximately half the rf energy as corresponding BURBOP-180 UR pulses. SORDOR-90 compared to time-optimal BURBOP-90 pulses lead to a reduction in pulse length and rf energy of approximately a factor of 1.8 .

Following the Böhlen and Bodenhausen concept [Bibr bib1.bibx9], which lately has found a revival in EPR spectroscopy [Bibr bib1.bibx23], the quadratic-phase SORDOR pulses can directly be used to replace pulses in any hard-pulse–delay sequence. The concept is schematically shown for the perfect echo sequence [Bibr bib1.bibx94] in Fig. [Fig Ch1.F3]: a train of 90 and 180 ${}^{\circ }$ pulses with delays ensures planar mixing conditions and therefore spin state conservation and in-phase to in-phase transfer. The sequence works well with hard pulses, but offset limitations sometimes require the application of broadband pulses. As the SORDOR pulses have matched quadratic-phase effective rotation axes at each optimised frequency offset, they directly act as universal rotation pulses, thereby enabling excitation as well as refocusing and mixing. Assuming that a SORDOR pulse sequence initially acts on polarisation, it will represent a broadband version of the hard-pulse sequence. The only difference to the hard-pulse version affects the acquisition, as the quadratic phase implies the use of a quadratic-phase correction (see below) or the application of a scaled SORDOR-180 pulse for phase refocusing. Simulations show that a matched SORDOR-180 pulse with rf amplitude and pulse length scaled by $\sqrt{2}$ and $1/\sqrt{2}$ , respectively, leads to effective refocusing of transverse magnetisation for acquisition without phase correction (see also the experimental section and Fig. [Fig Ch1.F4]e).

**Figure 3 Ch1.F3:**
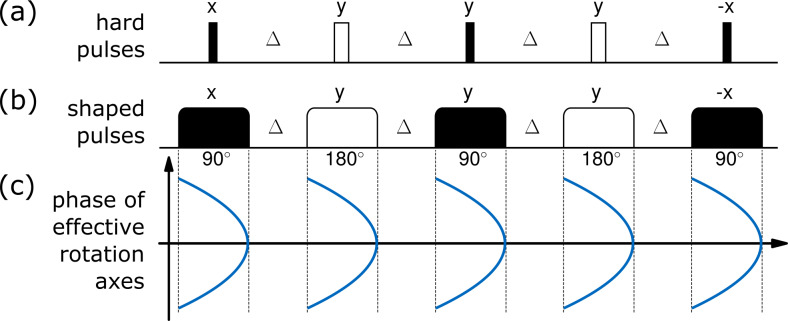
Schematic of the applicability of the Böhlen and Bodenhausen concept. **(a)** Hard-pulse perfect echo sequence; **(b)** corresponding shaped pulse scheme; **(c)** phase of effective rotation axes of SORDOR pulses with quadratic-phase dependence.

**Figure 4 Ch1.F4:**
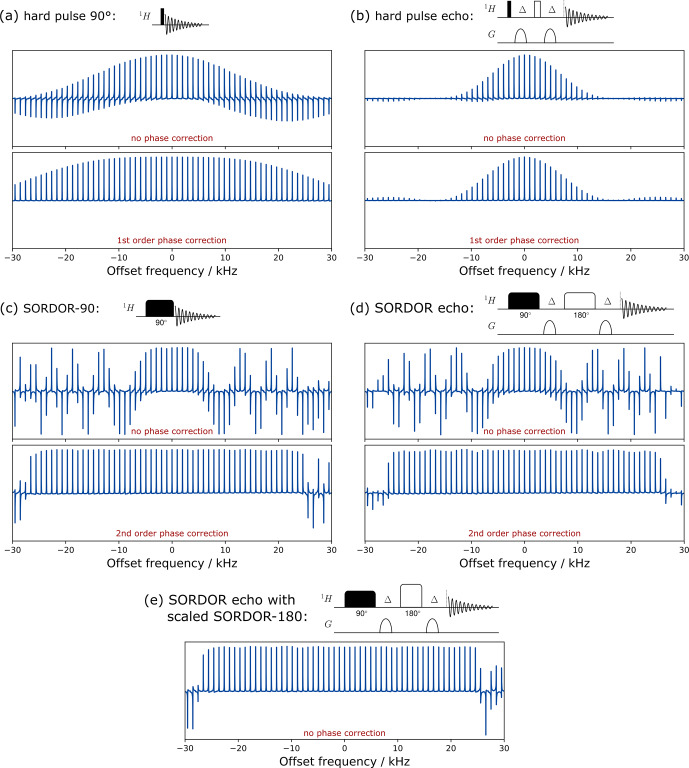
Experimental excitation profiles of a hard 90 ${}^{\circ }$ **(a)** and SORDOR-90 **(c)** pulse as well as a 90 ${}^{\circ }$ – Δ –180 ${}^{\circ }$ – Δ echo element for hard and SORDOR pulses **(b, d)** and a combination of SORDOR pulses with a scaled SORDOR-180 pulse **(e)**. Offset profiles of pulses are shown without phase correction and with linear (first-order, **a** and **b**) and quadratic (second-order, **c** and **d**) phase correction. The echo element with the SORDOR-180 scaled by $\sqrt{2}$ as described in the main text is self-refocusing and results in a perfect phase over the optimised bandwidth without phase correction **(e)**. The Bruker AU program used for second-order phase correction is given in the Supplement.

## 3 Experimental

The offset-dependent experimental performance of a single SORDOR-90 pulse and a combination SORDOR-90– Δ –SORDOR-180– Δ acquisition is shown in Fig. [Fig Ch1.F4] in comparison to corresponding hard-pulse elements. Applied gradients furthermore ensure that the desired coherence pathway is selected. While a single rectangular 90 ${}^{\circ }$ pulse results in a perfectly phaseable spectrum over large bandwidths, the 90 ${}^{\circ }$ – Δ –180 ${}^{\circ }$ – Δ sequence has a clearly reduced bandwidth. Also, the signal intensities are rapidly reduced with the offset for the two hard-pulse scenarios. The SORDOR-90 pulse, instead, produces a horribly looking offset dependence, which, however, results in nearly constant amplitude signals over the optimised bandwidth if a quadratic-phase correction is applied. Deviations of signal intensity over the optimised bandwidth are generally below ±2 %. Equally, the SORDOR pulse combination results in nicely refocused spectra after the second-order phase correction with deviations generally below ±4 %. A particularly interesting case for applications is shown in Fig. [Fig Ch1.F4]e, where the echo element is applied with a SORDOR-180 pulse with rf amplitude increased and pulse length decreased by $\sqrt{2}$ . In this case the transverse magnetisation is fully refocused, resulting in purely absorptive signals without any phase correction.

The applicability of the SORDOR pulse pair is demonstrated in a second example using ${}^{1}H$ - and ${}^{19}F$ -PROJECT experiments for the measurement of relaxation times without distortions due to coupling evolution [Bibr bib1.bibx94]. We applied the SORDOR-PROJECT sequence with two different SORDOR pairs and a hard-pulse PROJECT sequence to a doped $D_{2}O$ sample for comparing the effective relaxation rates of the residual HDO singlet. With decay times of 0.179 s (hard pulse), 0.178 s ( ±10 % B ${}_{1}$ -corrected SORDOR pair), and 0.169 s ( ±10 % B ${}_{1}$ -corrected SORDOR-pair), the relaxation measurements appear to give reproducible results, although the SORDOR-PROJECT experiment resulted in slightly stronger deviations from exponential decay (see the Supplement for data).

**Figure 5 Ch1.F5:**
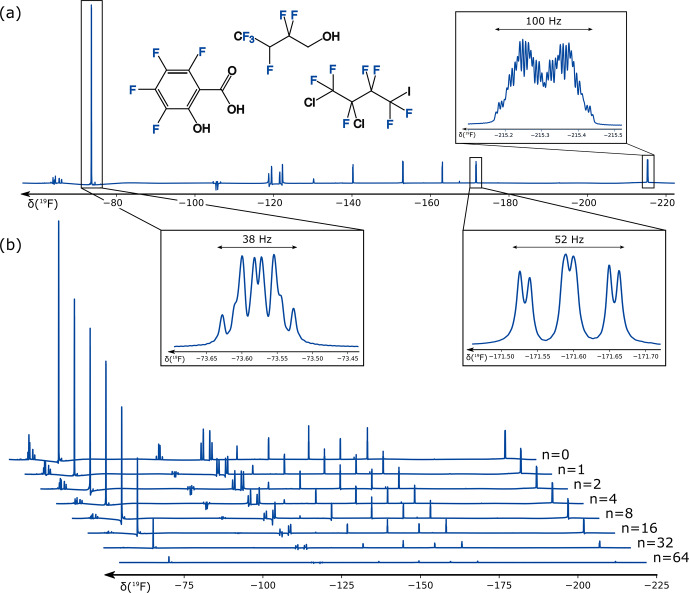
${}^{19}F$ -PROJECT experiment [Bibr bib1.bibx2] for relaxation measurement based on multiple SORDOR perfect echoes ( n=0 –64) with Δ=3 ms and SORDOR pulse lengths $t_{p}$ of 576 µ s each **(b)**. In addition, zooms for several large multiplets for the PROJECT spectrum with n=4 are given **(a)** with annotated multiplet widths.

For the ${}^{19}F$ -PROJECT experiment, corresponding spectra are shown in Fig. [Fig Ch1.F5] for a mixture of 1,1,1,2,3,3-hexafluoro-4-butanol, 1,2-dichloro-4-iodo-heptafluoro-n-butane and 1,2,3,4-tetrafluoro-salicylic acid in DMSO with ${}^{19}F$ chemical shifts ranging from -63 to -216 ppm. On the Avance III HD 400 MHz spectrometer used, this corresponds to a bandwidth of 58 kHz, which can be easily covered by the SORDOR pulse pair with rf amplitudes of 12.5 kHz. Clearly the relaxation of all signals can be easily followed using up to 64 perfect echoes with delays of Δ=3 ms and corresponding 576 µ s SORDOR-90 and SORDOR-180 pulses, which add with 432 µ s ( 75 %) per pulse to the effective delay. Thereby it is important to note that phase distortions due to coupling evolution are expected for a sum of couplings significantly larger than 1/Δ . We therefore had a closer look at the multiplets with large couplings that frequently appear in fluorinated compounds. Remarkably, most multiplets with widths up to 100 Hz still result in pure absorptive PROJECT spectra, demonstrating the robustness of the refocusing properties of the SORDOR approach. Only signals with apparent second-order artefacts at -63 to -68 , -104 to -109 , and -118 to -123 ppm show larger distortions. The spectra of all well-refocused signals result in mono-exponential decays from which decay times are easily derived. Spectra with apparent second-order artefacts are expected to show distorted decays (see the Supplement for decay plots and data). It should be noted that we also acquired hard-pulse ${}^{19}F$ -PROJECT experiments to validate data. However, resulting spectra – even when applied on-resonant – were heavily distorted and did not allow any extraction of decays. This is due to the insufficient bandwidth of accessible hard pulses, which leads to absorptive and dispersive contributions of in-phase as well as anti-phase magnetisation in the heavily coupled fluorine spin systems.

## 4 Discussion

Matched SORDOR-90 and SORDOR-180 pulses form a novel class of pulses with uniform rotation angles and a specific quadratic offset dependence of corresponding rotation axes. With such pulses the refocusing concepts of Böhlen and Bodenhausen for quadratic-phase pulses can generally be implemented. The SORDOR implementation allows the direct replacement of 90 and 180 ${}^{\circ }$ hard pulses in pulse–delay sequences.

As the simplest application, the concept allows the acquisition of 1D-type spectra with only an additional quadratic-phase correction. The AU program for Bruker spectrometers used for Fig. [Fig Ch1.F4] to perform a quadratic-phase correction is given in the Supplement. This quadratic-phase correction is always the same for a given pulse and can be determined once and then applied in all types of experiment with excitation using the same SORDOR-90 pulse.

In more complex pulse–delay experiments, like the PROJECT sequence shown in Fig. [Fig Ch1.F5] for a ${}^{19}F$ application, hard 90 ${}^{\circ }$ and 180 ${}^{\circ }$ pulses can directly be replaced by SORDOR-90 and SORDOR-180 pulses. If the last part of the sequence before acquisition consists of a Δ –180 ${}^{\circ }$ – Δ element, the quadratic-phase correction of the acquired FID may be compensated by using the scaled SORDOR-180 approach demonstrated in Fig. [Fig Ch1.F4]e. In this case the transverse magnetisation is fully refocused by applying scaling factors of $\sqrt{2}$ and $1/\sqrt{2}$ to rf amplitude and pulse length $t_{p}$ , respectively.

In 2D experiments, the indirect dimension can be evolved as in conventional hard-pulse experiments the offset dependencies for excitation and back transfer along z compensate each other.

A disadvantage of the SORDOR pulses arises in hetero-nuclear experiments when pulses on different nuclei need to be applied simultaneously. In these cases the same precautions apply that must also be taken, i.e. for adiabatic or other shaped pulses [Bibr bib1.bibx54]. Simultaneously applied hard pulses should be aligned to either the left- or right-hand side of the SORDOR pulses. Central application of pulses would need to be considered for a specific SORDOR pulse in use, e.g. with a toggling frame approach as demonstrated for selective pulses in INEPT-transfer elements [Bibr bib1.bibx46]. Simultaneous application with other shaped pulses may also lead to unexpected effects, as coupling evolution might take place during the pulses [Bibr bib1.bibx29]. Even if hard pulses are aligned left or right from the SORDOR pulses, offset-dependent coupling evolution cannot be avoided. However, in this case it is well-defined, and in the ${}^{1}H$ , ${}^{13}C$ correlation experiment it can be used like in Chirp applications to compensate for offset-dependent coupling-size compensation [Bibr bib1.bibx64].

In homo-nuclear experiments artefacts may arise from homo-nuclear coupling evolution during pulse shapes. Average Hamiltonian simulations on the non-adiabatic SORDOR pulse shapes used here, however, reveal that this effect is negligibly small, in contrast to adiabatic pulses with known considerable coupling effects (data not shown). Essentially only unavoidable artefacts for spin systems with close chemical shifts and second-order contributions must be taken into account.

It should be noted that the proposed approach considers that resonance frequencies during delays stay the same. Exchange effects and radiation damping will lead to offset changes and because of the offset-dependent rotation axes finally to distorted phases. Such distortions may be undesired or even helpful in the identification of e.g. exchange processes.

Finally, we would like to make the remark that the relatively short SORDOR pulses used in this study are compensated for ±5 % B ${}_{1}$ inhomogeneity with overall quality factors Φ>0.9999 . This quality factor corresponds to a quite good performance, as can be seen in Fig. [Fig Ch1.F4]. For probe heads exceeding the assumed ±5 % $B_{1}$ inhomogeneity, pulse performance might be reduced. We also experienced slightly reduced performance with high Q probe heads and correspondingly long switching times. To improve experimental performance, pulse shape optimisation eventually requires the adaptation of optimisation procedures with penalty functions regarding non-smooth waveforms [Bibr bib1.bibx44] or non-adiabaticity [Bibr bib1.bibx79], which will be subject to future efforts.

## 5 Conclusion

An implementation of the Böhlen and Bodenhausen concept using matched SORDOR-90 and SORDOR-180 pulse pairs is introduced. While SORDOR-180 pulses are equivalent to broadband inversion pulses with a defined, matched phase behaviour, corresponding SORDOR-90 pulses as introduced in [Bibr bib1.bibx45] belong to the B1 pulse class and allow uniform 90 ${}^{\circ }$ rotations around specific, quadratic offset-dependent rotation axes. The concept allows the direct replacement of hard pulses by their SORDOR equivalents, including 90 ${}^{\circ }$ mixing pulses, thereby enabling very broadband planar mixing and COSY-type experiments. As such, SORDOR pulse pairs act equivalently to universal rotation pulses but with considerably shorter pulse lengths and reduced rf energy. As an example, we introduced a broadband ${}^{19}F$ -PROJECT experiment with good refocusing and mixing results even for complex multiplets.

SORDOR-180 pulses afford the same rf energy as time-optimal inversion pulses like BIP [Bibr bib1.bibx90] or BIBOP [Bibr bib1.bibx56] pulses of the same quality. SORDOR-90 pulses require slightly more rf energy than time-optimal BEBOP excitation pulses [Bibr bib1.bibx56] but significantly less than corresponding BURBOP-90 time-optimal UR pulses [Bibr bib1.bibx59]. Therefore the SORDOR pulse pair represents the direct implementation of a broadband 90 ${}^{\circ }$ /180 ${}^{\circ }$ pulse–delay sequence with very low rf-energy deposition, with a reduction factor of rf requirements compared to conventional UR pulses slightly below 2 . With this improvement in rf usage, we expect the introduced approach to be useful in a variety of experiments like fast-pulsing 2Ds see e.g. and rf-limited imaging applications [Bibr bib1.bibx7].

## Supplement

10.5194/mr-3-53-2022-supplement The supplement related to this article is available online at: https://doi.org/10.5194/mr-3-53-2022-supplement.

## Data Availability

Pulses are available at https://www.ioc.kit.edu/luy/111.php (Haller et al., 2022), and codes can be found in the Supplement.

## References

[bib1.bibx1] Abramovich D, Vega S (1993). Derivation of Broadband and Narrowband Excitation Pulses Using the Floquet Formalism. J Magn Reson A.

[bib1.bibx2] Aguilar JA, Nilsson M, Bodenhausen G, Morris GA (2012). Spin echo NMR spectra without J modulation. Chem Commun.

[bib1.bibx3] Altenhof AR, Lindquist AW, Foster LDD, Holmes ST, Schurko RW (2019). On the use of frequency-swept pulses and pulses designed with optimal control theory for the acquisition of ultra-wideline NMR spectra. J Magn Reson.

[bib1.bibx4] Anand CK, Bain AD, Curtis AT, Nie Z (2012). Designing optimal universal pulses using second-order, large-scale, non-linear optimization. J Magn Reson.

[bib1.bibx5] Armstrong GS, Cano KE, Mandelshtam VA, Shaka AJ, Bendiak B (2004). Rapid 3D NMR using the filter diagonalization method: application to oligosaccharides derivatized with 
${}^{13}$
C-labeled acetyl groups. J Magn Reson.

[bib1.bibx6] Asami S, Kallies W, Gunther JC, Stavropoulou M, Glaser SJ, Sattler M (2018). Ultrashort Broadband Cooperative Pulses for Multidimensional Biomolecular NMR Experiments. Angew Chem Int Ed.

[bib1.bibx7] Barker PB, Golay X, Artemov D, Ouwerkerk R, Smith MA, Shaka AJ (2001). Broadband proton decoupling for in vivo brain spectroscopy in humans. Magn Reson Med.

[bib1.bibx8] Baum J, Tycko R, Pines A (1985). Broadband and adiabatic inversion of a two-level system by phase-modulated pulses. Phys Rev A.

[bib1.bibx9] Böhlen J-M, Rey M, Bodenhausen G (1989). Refocusing with chirped pulses for broadband excitation without phase dispersion. J Magn Reson.

[bib1.bibx10] Böhlen J-M, Burghardt I, Rey M, Bodenhausen G (1990). Frequency-modulated “Chirp” pulses for broadband inversion recovery in magnetic resonance. J Magn Reson.

[bib1.bibx11] Braun M, Glaser SJ (2010). Cooperative pulses. J Magn Reson.

[bib1.bibx12] Braun M, Glaser SJ (2014). Concurrently optimized cooperative pulses in robust quantum control: application to broadband Ramsey-type pulse sequence elements. New J Phys.

[bib1.bibx13] Brif C, Grace MD, Sarovar M, Young KC (2014). Exploring adiabatic quantum trajectories via optimal control. New J Phys.

[bib1.bibx14] Brown D (2011). Constant amplitude broadband refocusing pulses from numerical optimization. Magn Reson Chem.

[bib1.bibx15] Burghardt I, Böhlen J, Bodenhausen G (1990). Broadband multiple‐quantum nuclear magnetic resonance with frequency‐modulated “chirp” pulses: Applications to pairs of scalar‐coupled spin 
I=1/2
 nuclei. J Chem Phys.

[bib1.bibx16] Cano KE, Smith MA, Shaka AJ (2002). Adjustable, broadband, selective excitation with uniform phase. J Magn Reson.

[bib1.bibx17] Cho H, Pines A (1987). Iterative maps for broadband excitation of transverse coherence in two level systems. J Chem Phys.

[bib1.bibx18] Conolly S, Nishimura D, Macovski A (1986). Optimal control solutions to the magnetic resonance selective excitation problem. IEEE Trans Med Imaging.

[bib1.bibx19] Coote P, Bermel W, Arthanari H (2021). Optimization of phase dispersion enables broadband excitation without homonuclear coupling artifacts. J Magn Reson.

[bib1.bibx20] de Fouquieres P (2012). Implementing quantum gates by optimal control with doubly exponential convergence. Phys Rev Lett.

[bib1.bibx21] de Fouquieres P, Schirmer SG, Glaser SJ, Kuprov I (2011). Second order gradient ascent pulse engineering. J Magn Reson.

[bib1.bibx22] Doll A, Jeschke G (2014). Fourier-transform electron spin resonance with bandwidth-compensated chirp pulses. J Magn Reson.

[bib1.bibx23] Doll A, Jeschke G (2016). EPR-correlated dipolar spectroscopy by Q-band chirp SIFTER. Phys Chem Chem Phys.

[bib1.bibx24] Doll A, Jeschke G (2017). Double electron-electron resonance with multiple non-selective chirp refocusing. Phys Chem Chem Phys.

[bib1.bibx25] Doll A, Jeschke G (2017). Wideband frequency-swept excitation in pulsed EPR spectroscopy. J Magn Reson.

[bib1.bibx26] Ehni S, Luy B (2012). A systematic approach for optimizing the robustness of pulse sequence elements with respect to couplings, offsets, and B
${}_{1}$
-field inhomogeneities (COB). Magn Reson Chem.

[bib1.bibx27] Ehni S, Luy B (2013). BEBE
${}^{\mathrm{tr}}$
 and BUBI: 
J
-compensated concurrent shaped pulses for 
${}^{1}$
H-
${}^{13}$
C experiments. J Magn Reson.

[bib1.bibx28] Ehni S, Luy B (2014). Robust INEPT and refocused INEPT transfer with compensation of a wide range of couplings, offsets, and B
${}_{1}$
-field inhomogeneities (COB3). J Magn Reson.

[bib1.bibx29] Ehni S, Koos MRM, Reinsperger T, Haller JD, Goodwin DL, Luy B (12 September 2021). Concurrent J-Evolving Refocusing Pulses [preprint]. arXiv.

[bib1.bibx30] Emsley L, Bodenhausen G (1990). Gaussian pulse cascades: New analytical functions for rectangular selective inversion and in-phase excitation in NMR. Chem Phys Lett.

[bib1.bibx31] Emsley L, Bodenhausen G (1992). Optimization of shaped selective pulses for NMR using a quaternion description of their overall propagators. J Magn Reson.

[bib1.bibx32] Enders M, Görling B, Braun AB, Seltenreich JE, Reichenbach LF, Rissanen K, Nieger M, Luy B, Schepers U, Bräse S (2014). Cytotoxicity and NMR Studies of Platinum Complexes with Cyclooctadiene Ligands. Organometallics.

[bib1.bibx33] Enthart A, Freudenberger JC, Furrer J, Kessler H, Luy B (2008). The CLIP/CLAP-HSQC: pure absorptive spectra for the measurement of one-bond couplings. J Magn Reson.

[bib1.bibx34] Ewing B, Glaser SJ, Drobny GP (1990). Development and optimization of shaped NMR pulses for the study of coupled spin systems. Chem Phys.

[bib1.bibx35] Foroozandeh M (2020). Spin dynamics during chirped pulses: applications to homonuclear decoupling and broadband excitation. J Magn Reson.

[bib1.bibx36] Frankel J, Wilén J, Hansson Mild K (2018). Assessing Exposures to Magnetic Resonance Imaging's Complex Mixture of Magnetic Fields for In Vivo, In Vitro, and Epidemiologic Studies of Health Effects for Staff and Patients. Front Public Health.

[bib1.bibx37] Freeman R, Friedrich J, Wu XL (1988). A Pulse for All Seasons – Fourier-Transform Spectra without a Phase Gradient. J Magn Reson.

[bib1.bibx38] Garwood M, DelaBarre L (2001). The return of the frequency sweep: designing adiabatic pulses for contemporary NMR. J Magn Reson.

[bib1.bibx39] Garwood M, Ke Y (1991). Symmetric pulses to induce arbitrary flip angles with compensation for rf inhomogeneity and resonance offsets. J Magn Reson.

[bib1.bibx40] Gershenzon NI, Kobzar K, Luy B, Glaser SJ, Skinner TE (2007). Optimal control design of excitation pulses that accommodate relaxation. J Magn Reson.

[bib1.bibx41] Gershenzon NI, Skinner TE, Brutscher B, Khaneja N, Nimbalkar M, Luy B, Glaser SJ (2008). Linear phase slope in pulse design: application to coherence transfer. J Magn Reson.

[bib1.bibx42] Goodwin DL (2017). Advanced Optimal Control Methods for Spin Systems [PhD thesis].

[bib1.bibx43] Goodwin DL, Kuprov I (2015). Auxiliary matrix formalism for interaction representation transformations, optimal control, and spin relaxation theories. J Chem Phys.

[bib1.bibx44] Goodwin DL, Kuprov I (2016). Modified Newton-Raphson GRAPE methods for optimal control of spin systems. J Chem Phys.

[bib1.bibx45] Goodwin DL, Koos MRM, Luy B (2020). Second order phase dispersion by optimized rotation pulses. Phys Rev Research.

[bib1.bibx46] Haller JD, Bodor A, Luy B (2019). Real-time pure shift measurements for uniformly isotope-labeled molecules using X-selective BIRD homonuclear decoupling. J Magn Reson.

[bib1.bibx47] Haller JD, Goodwin DL, Luy B (2022). https://www.ioc.kit.edu/luy/111.php.

[bib1.bibx48] Hull WE, Croasmun WR, Carlson RMK (1994). Two-dimensional NMR Spectroscopy – Applications for Chemists and Biochemists.

[bib1.bibx49] Hwang TL, van Zijl PC, Garwood M (1997). Broadband adiabatic refocusing without phase distortion. J Magn Reson.

[bib1.bibx50] Jeschke G (2019). Quo vadis EPR?. J Magn Reson.

[bib1.bibx51] Jeschke G, Pribitzer S, Doll A (2015). Coherence Transfer by Passage Pulses in Electron Paramagnetic Resonance Spectroscopy. J Phys Chem B.

[bib1.bibx52] Keniry MA, Sanctuary BC (1992). Experimental-Verification of Composite Inversion Pulses Obtained by Series Expansion of the Offset Angle. J Magn Reson.

[bib1.bibx53] Khaneja N (2017). Chirp excitation. J Magn Reson.

[bib1.bibx54] Khaneja N (2018). Chirp mixing. Chem Phys Lett.

[bib1.bibx55] Khaneja N, Reiss T, Kehlet C, Schulte-Herbrüggen T, Glaser SJ (2005). Optimal control of coupled spin dynamics: design of NMR pulse sequences by gradient ascent algorithms. J Magn Reson.

[bib1.bibx56] Kobzar K, Skinner TE, Khaneja N, Glaser SJ, Luy B (2004). Exploring the limits of broadband excitation and inversion pulses. J Magn Reson.

[bib1.bibx57] Kobzar K, Luy B, Khaneja N, Glaser SJ (2005). Pattern pulses: design of arbitrary excitation profiles as a function of pulse amplitude and offset. J Magn Reson.

[bib1.bibx58] Kobzar K, Skinner TE, Khaneja N, Glaser SJ, Luy B (2008). Exploring the limits of broadband excitation and inversion: II. Rf-power optimized pulses. J Magn Reson.

[bib1.bibx59] Kobzar K, Ehni S, Skinner TE, Glaser SJ, Luy B (2012). Exploring the limits of broadband 90 degrees and 180 degrees universal rotation pulses. J Magn Reson.

[bib1.bibx60] Koos MRM, Feyrer H, Luy B (2015). Broadband excitation pulses with variable RF amplitude-dependent flip angle (RADFA). Magn Reson Chem.

[bib1.bibx61] Koos MRM, Feyrer H, Luy B (2017). Broadband RF-amplitude-dependent flip angle pulses with linear phase slope. Magn Reson Chem.

[bib1.bibx62] Kupče Ē, Freeman R (1994). Wideband Excitation with Polychromatic Pulses. J Magn Reson A.

[bib1.bibx63] Kupče Ē, Freeman R (1995). Adiabatic Pulses for Wideband Inversion and Broadband Decoupling. J Magn Reson A.

[bib1.bibx64] Kupče Ē, Freeman R (1997). Compensation for Spin–Spin Coupling Effects during Adiabatic Pulses. J Magn Reson.

[bib1.bibx65] Kupče Ē, Freeman R (2007). Fast multidimensional NMR by polarization sharing. Magn Reson Chem.

[bib1.bibx66] Levitt MH (1982). Symmetrical composite pulse sequences for NMR population inversion. I. Compensation of radiofrequency field inhomogeneity. J Magn Reson.

[bib1.bibx67] Levitt MH (1986). Composite pulses. Progr Nucl Magn Reson Spectrosc.

[bib1.bibx68] Levitt MH (1988). NMR Solvent Peak Suppression by Nonlinear Excitation. J Chem Phys.

[bib1.bibx69] Lingel A, Vulpetti A, Reinsperger T, Proudfoot A, Denay R, Frommlet A, Henry C, Hommel U, Gossert AD, Luy B, Frank AO (2020). Comprehensive and High-Throughput Exploration of Chemical Space Using Broadband 
${}^{19}$
F NMR-Based Screening. Angew Chem Int Ed.

[bib1.bibx70] Lurie DJ (1986). Numerical design of composite radiofrequency pulses. J Magn Reson.

[bib1.bibx71] Luy B, Kobzar K, Skinner TE, Khaneja N, Glaser SJ (2005). Construction of universal rotations from point-to-point transformations. J Magn Reson.

[bib1.bibx72] Mao J, Mareci TH, Scott KN, Andrew ER (1986). Selective inversion radiofrequency pulses by optimal control. J Magn Reson.

[bib1.bibx73] Moore J, Jankiewicz M, Anderson AW, Gore JC (2012). Evaluation of non-selective refocusing pulses for 7 T MRI. J Magn Reson.

[bib1.bibx74] Odedra S, Wimperis S (2012). Use of composite refocusing pulses to form spin echoes. J Magn Reson.

[bib1.bibx75] Ogura K, Terasawa H, Inagaki F (1996). Fully 
${}^{13}$
C-Refocused Multidimensional 
${}^{13}$
C-Edited Pulse Schemes Using Broadband Shaped Inversion and Refocusing Pulses. J Magn Reson B.

[bib1.bibx76] Poon CS, Henkelman RM (1992). 180
${}^{\circ }$
 refocusing pulses which are insensitive to static and radiofrequency field inhomogeneity. J Magn Reson.

[bib1.bibx77] Poon CS, Henkelman RM (1995). Robust Refocusing Pulses of Limited Power. J Magn Reson A.

[bib1.bibx78] Power JE, Foroozandeh M, Adams RW, Nilsson M, Coombes SR, Phillips AR, Morris GA (2016). Increasing the quantitative bandwidth of NMR measurements. Chem Commun.

[bib1.bibx79] Rosenfeld D, Zur Y (1996). Design of adiabatic selective pulses using optimal control theory. Magn Reson Med.

[bib1.bibx80] Schulze-Sünninghausen D, Becker J, Luy B (2014). Rapid heteronuclear single quantum correlation NMR spectra at natural abundance. J Am Chem Soc.

[bib1.bibx81] Schulze-Sünninghausen D, Becker J, Koos MRM, Luy B (2017). Improvements, extensions, and practical aspects of rapid ASAP-HSQC and ALSOFAST-HSQC pulse sequences for studying small molecules at natural abundance. J Magn Reson.

[bib1.bibx82] Shaka AJ (1985). Composite pulses for ultra-broadband spin inversion. Chem Phys Lett.

[bib1.bibx83] Shaka AJ, Freeman R (1983). Composite pulses with dual compensation. J Magn Reson.

[bib1.bibx84] Shaka AJ, Pines A (1987). Symmetric phase-alternating composite pulses. J Magn Reson.

[bib1.bibx85] Skinner TE, Reiss TO, Luy B, Khaneja N, Glaser SJ (2003). Application of optimal control theory to the design of broadband excitation pulses for high-resolution NMR. J Magn Reson.

[bib1.bibx86] Skinner TE, Reiss TO, Luy B, Khaneja N, Glaser SJ (2004). Reducing the duration of broadband excitation pulses using optimal control with limited RF amplitude. J Magn Reson.

[bib1.bibx87] Skinner TE, Reiss TO, Luy B, Khaneja N, Glaser SJ (2005). Tailoring the optimal control cost function to a desired output: application to minimizing phase errors in short broadband excitation pulses. J Magn Reson.

[bib1.bibx88] Skinner TE, Kobzar K, Luy B, Bendall MR, Bermel W, Khaneja N, Glaser SJ (2006). Optimal control design of constant amplitude phase-modulated pulses: application to calibration-free broadband excitation. J Magn Reson.

[bib1.bibx89] Skinner TE, Gershenzon NI, Nimbalkar M, Bermel W, Luy B, Glaser SJ (2012). New strategies for designing robust universal rotation pulses: application to broadband refocusing at low power. J Magn Reson.

[bib1.bibx90] Smith MA, Hu H, Shaka AJ (2001). Improved broadband inversion performance for NMR in liquids. J Magn Reson.

[bib1.bibx91] Spindler PE, Zhang Y, Endeward B, Gershernzon N, Skinner TE, Glaser SJ, Prisner TF (2012). Shaped optimal control pulses for increased excitation bandwidth in EPR. J Magn Reson.

[bib1.bibx92] Spindler PE, Waclawska I, Endeward B, Plackmeyer J, Ziegler C, Prisner TF (2015). Carr-Purcell Pulsed Electron Double Resonance with Shaped Inversion Pulses. J Phys Chem Lett.

[bib1.bibx93] Spindler PE, Schöps P, Kallies W, Glaser SJ, Prisner TF (2017). Perspectives of shaped pulses for EPR spectroscopy. J Magn Reson.

[bib1.bibx94] Takegoshi K, Ogura K, Hikichi K (1989). A perfect spin echo in a weakly homonuclear J-coupled two spin-12 system. J Magn Reson.

[bib1.bibx95] Tal A, Frydman L (2010). Single-scan multidimensional magnetic resonance. Prog Nucl Magn Reson Spectrosc.

[bib1.bibx96] Tycko R, Cho HM, Schneider E, Pines A (1985). Composite pulses without phase distortion. J Magn Reson.

[bib1.bibx97] Tycko R, Pines A, Guckenheimer J (1985). Fixed point theory of iterative excitation schemes in NMR. J Chem Phys.

[bib1.bibx98] Tzvetkova P, Simova S, Luy B (2007). P.E.HSQC: a simple experiment for simultaneous and sign-sensitive measurement of (
${}^{1}J_{\mathrm{CH}}+D_{\mathrm{CH}}$
) and (
${}^{2}J_{\mathrm{HH}}+D_{\mathrm{HH}}$
) couplings. J Magn Reson.

[bib1.bibx99] Van Loan C (1978). Computing integrals involving the matrix exponential. IEEE Trans Automat Contr.

[bib1.bibx100] Warren WS (1984). Effects of arbitrary laser or NMR pulse shapes on population inversion and coherence. J Chem Phys.

[bib1.bibx101] Wimperis S (1991). Iterative schemes for phase-distortionless composite 180
${}^{\circ }$
 pulses. J Magn Reson.

[bib1.bibx102] Wimperis S (1994). Broadband, Narrowband, and Passband Composite Pulses for Use in Advanced NMR Experiments. J Magn Reson A.

[bib1.bibx103] Zax DB, Goelman G, Vega S (1988). Amplitude-Modulated Composite Pulses. J Magn Reson.

[bib1.bibx104] Zwahlen C, Legault P, Vincent SJF, Greenblatt J, Konrat R, Kay LE (1997). Methods for Measurement of Intermolecular NOEs by Multinuclear NMR Spectroscopy: Application to a Bacteriophage 
λ
 N-Peptide/boxB RNA Complex. J Am Chem Soc.

