# Supplementary material for: SORDOR pulses: expansion of the Böhlen–Bodenhausen scheme for low-power broadband magnetic resonance
Source: Magn Reson (Gott). 2022 Apr 27;3(1):53–63. doi: 10.5194/mr-3-53-2022 (PMC10539771; doi:10.5194/mr-3-53-2022)
Supplement: The supplement related to this article is available online at: https://doi.org/10.5194/mr-3-53-2022-supplement. [file mr-3-53-supplement.pdf]

Supplement of Magn. Reson., 3, 53–63, 2022
https://doi.org/10.5194/mr-3-53-2022-supplement
© Author(s) 2022. CC BY 4.0 License.

Open Access
MAGNETIC RESONANCE

Supplement of

SORDOR pulses: expansion of the Böhlen–Bodenhausen scheme for low-power broadband magnetic resonance

Jens D. Haller et al.

Correspondence to: David L. Goodwin (david.goodwin@partner.kit.edu) and Burkhard Luy (burkhard.luy@kit.edu)

The copyright of individual parts of the supplement might differ from the article licence.

Contents

Classification of Shaped Pulses 2

Exponential Fits: Figure 5 4

Exponential Fits: Comparison to On-Resonant Hard Pulse 5

SORDOR pulse shapes 6

- **SORDOR-90 (720 μs, 50kHz bandwidth, 10kHz RF-ampl., ±5% compensation of B1-inhomog.)** 6
- **SORDOR-180 (720 μs, 50kHz bandwidth, 10kHz RF-ampl., ±5% compensation of B1-inhomog.)** 6
- **SORDOR-90 (720 μs, 50kHz bandwidth, 10kHz RF-ampl., ±10% compensation of B1-inhomog.)** 6
- **SORDOR-180 (720 μs, 50kHz bandwidth, 10kHz RF-ampl., ±10% compensation of B1-inhomog.)** 6

Pulse program for SORDOR 1D 7

Pulse program for SORDOR Echo 7

Pulse program for ^{19}F-PROJECT using SORDOR 8

Python scripts for second order phase correction 9

- **Second order phase correction for 1D** 9
- **Second order phase correction for ^{19}F-PROJECT** 10

Classification of Shaped Pulses

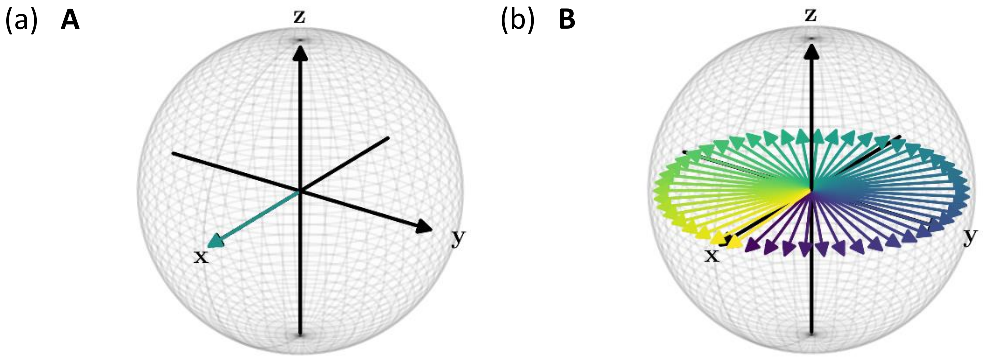

Figure S1: Classification of 180° shaped pulses following Levitt (1986). Effective rotations are illustrated as normalized vectors for various offsets.

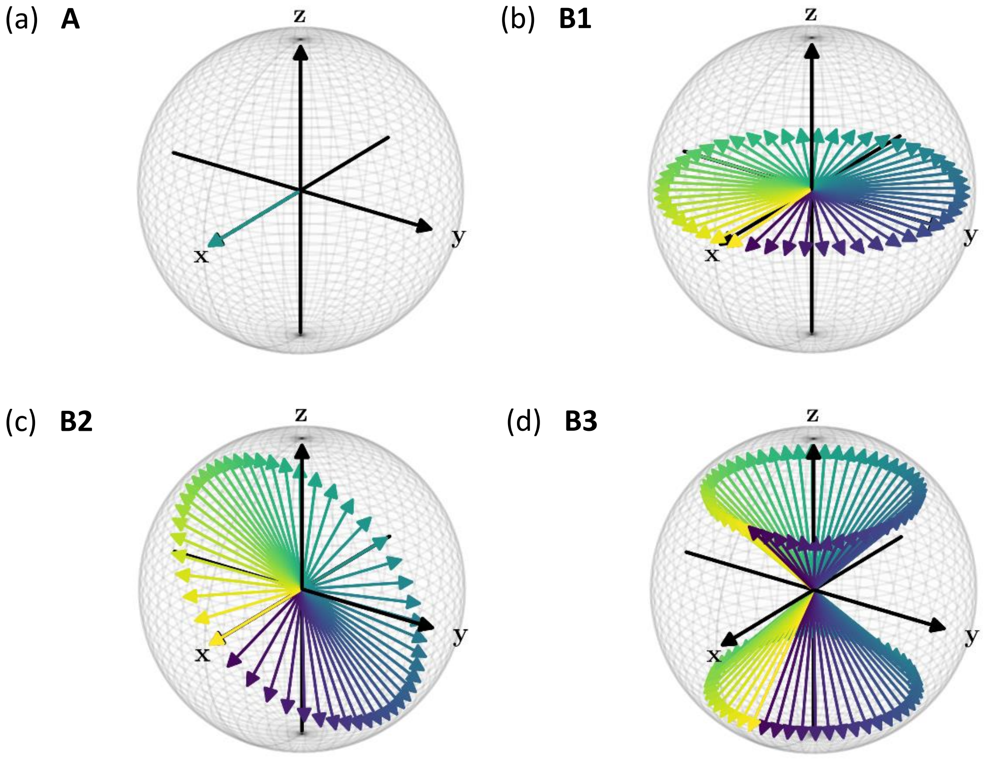

Figure S2: Classification of 90° shaped pulses following M. H. Levitt (1986). Effective rotations are illustrated as normalized vectors for various offsets.

Shaped pulses are composed of numerous much shorter pulses that can be modulated in phase and amplitude. The effective propagator U_{eff} of a shaped pulse, consisting of n steps, can hence be described by a piece-wise time-independent propagation:

$$U_{eff} = U_n \dots U_1.$$

Such a propagator U_{eff} represents an “effective rotation” that can be used for pulse shape classification as illustrated in Figure S1 and S2 for different 180° and 90° pulses, respectively.

The most versatile class **A** of 180° pulses is shown in Fig S1 (a) where a rotation about the x-axis is achieved for all considered offsets. Shaped 180° pulses of class **A** are typically referred to as “refocusing pulse” or “universal rotations” (UR) with $U_{eff} = U_{180x}$. Shaped 180° pulses of class **B**, on the other hand, induce phase shifts for transverse components but can be used as an inversion of the z-component – the effective rotations are shown in Fig S1 (b). These shaped 180° pulses are generally referred to as “point-to-point” or “inversion pulses” and $U_{eff} = U_\varphi U_{180x} U_\varphi^\dagger$ where U_φ corresponds to an arbitrary z-rotation.

For shaped 90° pulses a more distinctive classification is required as shown in Fig S2. Shaped 90° pulses of class **A** induce a 90° rotation about a single axis for all considered offsets and are, hence, also referred to as “universal rotations” where $U_{eff} = U_{90x}$ in Fig S2 (a). Effective rotations for shaped 90° pulses of class **B1** are illustrated in Fig S2 (b) where offset-dependent rotation axes are distributed in the transverse plane with $U_{eff} = U_\varphi U_{90x} U_\varphi^\dagger$. With respect to SORDOR-90 pulses, it is crucial to note that this class of 90° pulses can be considered universal rotations where an offset dependent phase is acquired. Pulse shapes of class **B2**, on the other hand, can only be used to transfer a single component of magnetization. Therefore, **B2**-pulses are typically used to excite z-magnetization to the transverse plane with defined phase (e.g. to $\hat{I}_y$) as shown in Fig S2 (c) with $U_{eff} \hat{I}_z U_{eff}^\dagger = \hat{I}_y$. It is noteworthy, that for these so-called “point-to-point” or “excitation pulses” the effective rotations are in a tilted plane. This is based on the fact that a transfer from $\hat{I}_z$ to $\hat{I}_y$ can be obtained e.g. by a 90° rotation about x but also by a 270° degree rotation about -x and further by a 180° rotation about the axes in the intersection of the tilted and the yz-plane. An excitation of z-magnetization to a state with undefined phase can be obtained from shaped pulses of class **B3** where $U_{eff} \hat{I}_z U_{eff}^\dagger = U_\varphi \hat{I}_y U_\varphi^\dagger$ and U_φ corresponds to an arbitrary z-rotation. In Fig S2 (d) certain effective rotation axes are shown but in principle all axes in between the two cones are thinkable.

For a „matched“ pulse pair of a SORDOR-180 (**B**) and a SORDOR-90 (**B1**) the effective rotations are distributed identically in the transverse plane or in other words U_φ is the same for the SORDOR-180 and the SORDOR-90. For this reason the pulse pair can be used as universal rotations with quadratic phase distribution.

Exponential Fits: Figure 5

Signals of Figure 5 were extracted and fitted to an exponential decay using python. Due to strong coupling, CF_2 groups exhibit phase distortions and exponential fits show large residuals (red box).

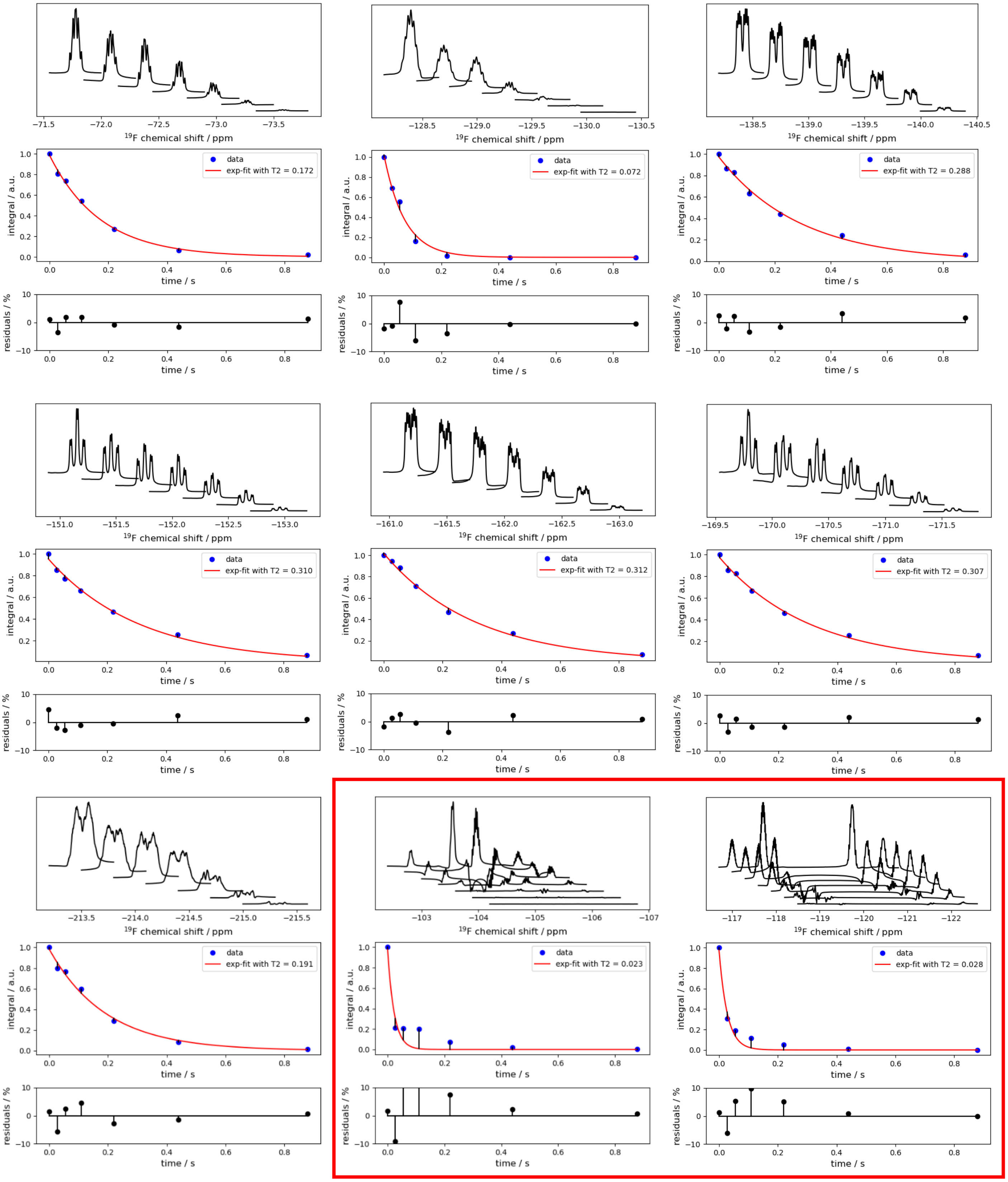

Exponential Fits: Comparison to On-Resonant Hard Pulse

Exponential decays of the PROJECT experiment using SORDOR pulse pairs are compared to on-resonant hard pulses for the Bruker "doped water" standard sample (0.1mg/ml $GdCl_3$, 1% H_2O and 0.1% ^{13}C-labeled CH_3OH in D_2O). Since the water signal is not subject to coupling evolution the decay rate does not depend on the mixing for *J* refocusing induced by PROJECT and it is possible to estimate that SORDOR pulse imperfections have only a minor influence on the relaxation measurement. SORDOR pulses are also used on-resonant, but a much larger bandwidth is expected (as shown in Figure 5 of main text).

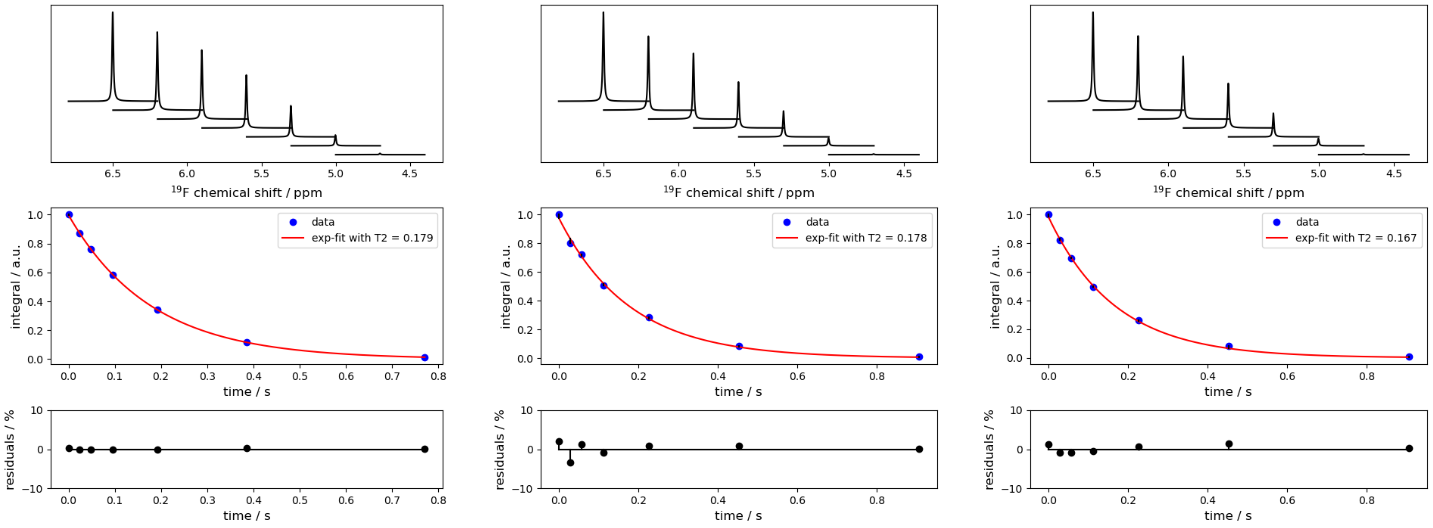

Figure S3: Exponential fits of PROJECT using on-resonant hard pulses (left), SORDOR pulse pairs with ±5% (middle) and ±10% (right) compensation of B_1-inhomogeneity (right) are illustrated for the Bruker "doped water" standard sample.

SORDOR pulse shapes

Pulse shapes are found in the available archive: „SORDOR_pulse_shapes.zip“

SORDOR-90 (720 µs, 50kHz bandwidth, 10kHz RF-ampl., ±5% compensation of B1-inhomog.)

File name: SORDOR_90_720us_RF10_BW50_pm5

SORDOR-180 (720 µs, 50kHz bandwidth, 10kHz RF-ampl., ±5% compensation of B1-inhomog.)

File name: SORDOR_180_720us_RF10_BW50_pm5

SORDOR-90 (720 µs, 50kHz bandwidth, 10kHz RF-ampl., ±10% compensation of B1-inhomog.)

File name: SORDOR_90_720us_RF10_BW50_pm10

SORDOR-180 (720 µs, 50kHz bandwidth, 10kHz RF-ampl., ±10% compensation of B1-inhomog.)

File name: SORDOR_180_720us_RF10_BW50_pm10

Pulse program for SORDOR 1D

```
;1D using shaped pulse

#include <Avance.incl>

1 ze
 30m pl=0[Watt]:f1
2 50m
   d1
  (p11:sp11 ph1):f1
  go=2 ph31
  50m mc #0 to 2 F0(zd)
exit

ph1=0 2 2 0 1 3 3 1
ph31=0 2 2 0 1 3 3 1


;pl1 : f1 channel - power level for pulse (default)
;p1 : f1 channel -  high power pulse
;d1 : relaxation delay; 1-5 * T1
;ns: 1 * n, total number of scans: NS * TD0
```

Pulse program for SORDOR Echo

```
;1D gradient echo using shaped pulses

#include <Avance.incl>
#include <Grad.incl>
#include <Delay.incl>

"spoffs12=spoffs11"

1 ze
 30m pl=0[Watt]:f1
2 50m
  d1
  50u UNBLKGRAD
  (p11:sp11 ph1):f1 ;SORDOR-90
  20u
  p16:gp3
  d16
  (p12:sp12 ph1):f1 ;SORDOR-180
  p16:gp3
  d16
  20u BLKGRAMP
  go=2 ph31
  50m mc #0 to 2 F0(zd)
  20u LOCKH_OFF
exit

ph1=0 2 2 0 1 3 3 1
ph31=0 2 2 0 1 3 3 1

;pl1 : f1 channel - power level for pulse (default)
;p1 : f1 channel -  high power pulse
;d1 : relaxation delay; 1-5 * T1
;ns: 1 * n, total number of scans: NS * TD0
```

Pulse program for ^{19}F-PROJECT using SORDOR

```
;19F-PROJECT using SORDOR pulses

#include <Avance.incl>

"d11=30m"

1 ze
2 d1 pl=0[Watt]:f1 rpp3 rpp4
  (p11:sp11 ph1):f1  ;SORDOR-90

3 d20
  (p12:sp12 ph2+ph3):f1  ;SORDOR-180
  d20
  (p11:sp11 ph2+ph3):f1 ;SORDOR-90
  d20
  (p12:sp12 ph2+ph3):f1  ;SORDOR-180
  d20 ipp3 ipp4
  lo to 3 times c

4 d20 dpp4
  (p12:sp12 ph2+ph4):f1  ;SORDOR-180
  d20
  (p11:sp11 ph2+ph4):f1  ;SORDOR-90
  d20
  (p12:sp12 ph2+ph4):f1  ;SORDOR-180
  d20
  lo to 4 times c

  ;(p13:sp13 ph1):f1  ;scaled SORDOR-180 for phase compensation (optional)

  go=2 ph31
  d11 wr #0 if #0 ivc
  lo to 1 times td1
exit


ph1=0 0 2 2 1 1 3 3
ph2=1 3 1 3 0 2 0 2

ph3=0 0 2 2
ph4=0 0 2 2

ph31=0 0 2 2 1 1 3 3

;pl1 : f1 channel - power level for pulse (default)
;p1 : f1 channel -  90 degree high power pulse
;p2 : f1 channel - 180 degree high power pulse
;d1 : relaxation delay; 1-5 * T1
;d11: delay for disk I/O                             [30 msec]
;d20: fixed echo time to allow elimination of diffusion
;     and J-mod. effects
;vc : variable loop counter, taken from vc-list

;td1: number of experiments = number of values in vc-list
;define VCLIST
;this pulse program produces a ser-file (PARMOD = 2D)
;d20: PROJECT delay
;vc : vc should contain even numbers to provide
;     for cancellation of 180 degree pulse errors
```

Python scripts for second order phase correction

In order to execute python scripts in Topspin, please run the „edpy“ command in Topspin and create new python scripts called pk2.py (1D version) and pk22D.py (pseudo-2D version). Copy and paste the subsequent code to the created scripts and execute from the Topspin command line.
For a 1D, please run „pk2 ϵ“ (e.g. „pk2 3.65“), where ϵ is the value for second order phase correction and the phase φ is calculated from

$$\varphi(\nu,\epsilon) = 2\pi \cdot \left(\frac{2\nu}{sw}\right)^2 \cdot \epsilon,$$

where sw is the spectral width and ν is the frequency offset. Setting the spectral width to the band-width covered by SORDOR pulses can facilitate the procedure.
For a pseudo-2D (e.g. ^{19}F-PROJECT), the second order phase correction is applied successively and the pseudo-2D is stored as multiple 1Ds. The script is executed with „pk22D ϵ `procno`“, where `procno` is the processing number of the *first* 1D spectrum and following 1Ds are stored in ascending numbers.

Second order phase correction for 1D

```
# call script like: pk2 "value for pk"
# example: pk2 3.65

import sys
import math

slope = float(sys.argv[1])

spect_real = GETPROCDATA(-500, 500)
spect_imag = GETPROCDATA(-500, 500, dataconst.PROCDATA_IMAG)

for i in range(len(spect_real)):
	phase=(i-len(spect_real)/2)**2 *8*math.pi*slope/len(spect_real)**2

	dummyx=spect_real[i]*math.cos(phase) - spect_imag[i]*math.sin(phase)
	dummyy=spect_real[i]*math.sin(phase) + spect_imag[i]*math.cos(phase)

	spect_real[i]=dummyx
	spect_imag[i]=dummyy

SAVE_ARRAY_AS_1R1I(spect_real,spect_imag)
```

Second order phase correction for ^{19}F-PROJECT

```
# second order phase correction on 2D
# it will create multiple 1Ds starting at a certain "procno"
# call script like: pk22D "value for pk" "procno"
# example: pk22D 3.65 900

import sys, math

td = int(GETPAR2("s1 TD"))
curdat = CURDATA()
slope = float(sys.argv[1])

for i in range(td):
	RSR(str(i+1), str(int(sys.argv[2])+i))

	#_______________________________________________
	# pk2.py
	spect_real = GETPROCDATA(-500, 500)
	spect_imag = GETPROCDATA(-500, 500,dataconst.PROCDATA_IMAG)

	for i in range(len(spect_real)):
		phase=(i-len(spect_real)/2)**2 *8*math.pi*slope/len(spect_real)**2

		dummyx=spect_real[i]*math.cos(phase) - spect_imag[i]*math.sin(phase)
		dummyy=spect_real[i]*math.sin(phase) + spect_imag[i]*math.cos(phase)

		spect_real[i]=dummyx
		spect_imag[i]=dummyy

	SAVE_ARRAY_AS_1R1I(spect_real,spect_imag)
	#_______________________________________________

	RE(curdat)
```
